# ﻿Three new species of *Peroneutypa* (Diatrypaceae, Xylariales) and a first record of *Eutypacamelliae* in China with updated description

**DOI:** 10.3897/mycokeys.114.145312

**Published:** 2025-02-28

**Authors:** Xinying Mao, Kamran Habib, Rizwana Zulfiqar, Hongde Yang, Yingqian Kang

**Affiliations:** 1 Key Laboratory of Microbiome and Infectious Disease Prevention and Control of Guizhou & Key Laboratory of Environmental Pollution Monitoring and Disease Control, Ministry of Education of Guizhou & School of Basic Medical Science & Institution of One Health Research, Guizhou Medical University, Guiyang, 550025, China Guizhou Medical University Guiyang China; 2 The High Efficacy Application of Natural Medicinal Resources Engineering Center of Guizhou Province (The Key Laboratory of Optimal Utilization of Natural Medicine Resources), School of Pharmaceutical Sciences, Guizhou Medical University, Gui’an New District, 561113, China Guizhou Medical University Gui’an New District China; 3 Institute of Botany, Fungal Biology and Systematics Lab, University of the Punjab, Quaid-e-Azam Campus, Lahore, 54590, Pakistan University of the Punjab Lahore Pakistan; 4 Centre for Yunnan Plateau Biological Resources Protection and Utilization, College of Biological Resource and Food Engineering, Qujing Normal University, Qujing 655011, Yunnan, China Qujing Normal University Qujing China

**Keywords:** 3 new species, diatrypaceous fungi, fungal systematics, Karst environment

## Abstract

Diatrypaceae is a diverse family with a worldwide distribution, occurring on a wide range of hosts in terrestrial and marine environments, some of which are important plant pathogens. During a survey of ascomycete diversity in Guizhou Province, China, three new taxa within *Peroneutypa* are proposed based on morphological comparisons and phylogenetic analyses of combined ITS and *tub2* sequences data. The newly proposed species are *Peroneutypaguizhouensis*, *P.wanfenglinensis* and *P.zhujiashanesis*. In addition, *Eutypacamelliae* was recorded for the first time from China, with an updated description. Detailed morphological descriptions, illustrations, comparative analyses, and a tabular comparison of the new species with related and similar taxa are provided.

## ﻿Introduction

Diatrypaceae is a diverse and ecologically important family of higher ascomycetes within the order Xylariales, inhabiting a wide variety of hosts in both terrestrial and marine environments worldwide (Chlebicki 1986; Glawe and Jacobs 1987; Carmarán and Romero 1992; Carmarán et al. 2006; Trouillas and Gubler 2010; de Almeida et al. 2016; Li et al. 2023). The members of Diatrypaceae are distributed worldwide, can be found on a wide range of plant species, from economically important crops to forest trees with different life modes, functioning as saprobes, pathogens, and endophytes (Vasilyeva and Ma 2014; Dayarathne et al. 2016; Mayorquin et al. 2016; Senwanna et al. 2017; Hyde et al. 2020; Konta et al. 2020). Members of the Diatrypaceae are characterized by black or dark brown, immersed or erumpent, eustromatic or pseudostromatic stromata, 8-spored or polysporous asci with a very long pedicel and J-/J+ apical apparatus, hyaline to light brown allantoid ascospores, and a libertella-like asexual morph (Senanayake et al. 2015; Wijayawardene et al. 2017).

In Wijayawardene et al. (2022) 22 genera within Diatrypaceae have been documented. However, recent taxonomic advances have led to the addition of three more genera, *Alloeutypa, Pseudoeutypa*, and *Stromatolinea*, bringing the total to 25 recognized genera (Ma et al. 2023; Habib et al. 2024; Zhang et al. 2024). Collectively, these genera encompass approximately 1000 species. Most of the species belong to *Cryptosphaeria*, *Diatrype*, *Diatrypella*, *Eutypa*, and *Eutypella* (Wijayawardene et al. 2022) and are polyphyletic. The polyphyletic nature of these genera arises primarily from the phenotypic plasticity and anatomical similarities observed within Diatrypaceae, where stromatal characteristics are highly variable and often unreliable for clear species delineation (Zhu et al. 2021; Li et al. 2023; Habib et al. 2024). Previous studies have highlighted the challenges in distinguishing members of Diatrypaceae based on morphology alone, with many taxa still lacking molecular data. This absence of molecular data complicates the classification of Diatrypaceae (de Almeida et al. 2016; Shang et al. 2018; Du et al. 2022). Similar difficulties are evident in *Peroneutypa*. Currently, 38 species of *Peroneutypa* are listed in Species Fungorum (https://www.speciesfungorum.org), but molecular data are available for only 20 of these species.

*Peroneutypa* was established by Berlese (1902), but no type species was designated at the time. Later, Rappaz (1987) proposed *P.bellula* as the type species and considered the genus as a synonym of *Eutypella*. Carmarán et al. (2006) reinstated *Peroneutypa* as a distinct genus based on its ascal type and phylogenetic analyses. *Peroneutypa* is distinguished by its urn-shaped asci, which have a truncate apex and are wider in the middle, where ascospores tend to cluster. In contrast, *Eutypella* features spindle-shaped asci with ascospores that cluster and swell in the upper portion (Carmarán et al. 2006). *Peroneutypa* species are characterized by valsoid stroma, ascomata with long prominent necks, sessile to long-stalked asci, with truncate apices and allantoid hyaline or yellowish ascospores (Carmarán et al. 2006; Vasilyeva and Rogers 2010; Shang et al. 2017). Species of the genus are known as saprobes or pathogens and are widely distributed in terrestrial and marine habitats (Shang et al. 2017; Dayarathne et al. 2020; Du et al. 2022)

In a study focused on the diversity of ascomycetes in Guizhou, China, we identified several Diatrypaceae specimens that did not match any known species. To clarify their taxonomic status, we performed phylogenetic analyses using the internal transcribed spacer (ITS) and β-tubulin (*tub2*) gene regions. These analyses led to the discovery of three new species belonging to *Peroneutypa*. We present a brief diagnosis, descriptions, images, and phylogenetic placement of these new species.

## ﻿Materials and methods

### ﻿Collection and isolation

Ascomycetous fungi associated with decayed branches and twigs of various plants were collected during surveys conducted in Guizhou Province, China. All related habitat information, including details about elevation, climatic conditions, and geographical features, was recorded. The photos of the collected materials were taken using a Canon G15 camera (Canon Corporation, Tokyo, Japan). Materials were placed in paper bags and were taken to the lab for examination. To preserve the freshness of the specimens, they were dried at room temperature. Fungal isolates were obtained through single spore isolation, following the method described by Senanayake et al. (2020). Spores were observed under a Stereo Zoom microscope and transferred to potato dextrose agar (PDA; 39 g/L in distilled water, Difco potato dextrose). Cultures were incubated at 25–30 °C for 1–4 weeks with regular observations. Cultural characteristics, including mycelial color, shape, texture, and growth rate, were documented under normal light conditions.

Herbarium specimens were deposited in the Cryptogams Herbarium of the Kunming Institute of Botany, the Chinese Academy of Sciences (KUN-HKAS), and the Guizhou Provincial Key Laboratory of Agricultural Biotechnology (GZAAS).

### ﻿Morphological study

Macroscopic characteristics were observed under an Olympus SZ61 stereomicroscope and photographed with a Canon 700D digital camera fitted to a light microscope (Nikon Ni). The morphological characteristics of specimens were examined, and photomicrographs were taken as described in Senanayake et al. (2020). Materials were mounted in water for anatomical examination, and Melzer’s reagent was used where necessary. More than 30 ascospores and 30 asci were measured using the Tarosoft ® image framework (v. 0.9.0.7). Images were arranged using Adobe Photoshop CS6 (Adobe Systems, USA).

### ﻿DNA extraction, PCR amplification, and sequencing

Genomic DNA was extracted from mycelium sourced from colonies cultured on PDA after 1–2 weeks at 25 °C, using the BIOMIGA Fungal gDNA Isolation Kit (BIOMIGA, Hangzhou City, Zhejiang Province, China). The DNA samples were stored at –20 °C. Internal transcribed spacers (ITS), and β-tubulin (*tub2*), were amplified by PCR with primers ITS1/ITS4 (White et al. 1990; Gardes and Bruns 1993), and Bt2a / Bt2b (Glass and Donaldson 1995; O’Donnell and Cigelnik 1997), respectively. The components of a 25 μL volume PCR mixture was: 9.5 μL of double distilled water, 12.5 μL of PCR Master Mix, 1 μL of each primer and 1 μL of template DNA. The PCR amplification was conducted as reported by Samarakoon et al. (2022). Qualified PCR products were checked through 1.5% agarose gel electrophoresis stained with GoldenView, and sent to Qingke Biotech Chongqing, China, for sequencing.

### ﻿Phylogenetic analyses

The newly generated forward and reverse sequences from this study were assembled in the BioEdit v. 7.0.5 (Hall 1999) then were subjected to BLASTn search against the GenBank nucleotide database at the National Center for Biotechnology Information (NCBI) to identify closely related sequences. Sequence data of related taxa were obtained from previous publications (Long et al. 2021; Zhu et al. 2021; Li et al. 2023) and downloaded from the GenBank database (Table 1). The sequences were aligned using MAFFT v.7 online web server (Katoh et al. 2019) under default settings. Alignment was adjusted manually using BioEdit v.7.0.5.3 (Hall 1999) where necessary. The combined sequence data was used to perform maximum likelihood (ML) and Bayesian inference analysis (BI). The ML analysis was implemented in RAxML v.8.2.12 using the GTR substitution model with 1,000 bootstrap replicates (Stamatakis 2014). Bayesian inference analysis was conducted in MrBayes v. 3.2.2 (Ronquist et al. 2012) online, with Markov chain Monte Carlo (MCMC) sampling in MrBayes v.3.2.2 (Ronquist et al. 2012) used to calculate posterior probabilities (PP). Six simultaneous Markov chains were run for 1,000,000 generations, and trees were sampled every 1,000^th^ generation. The convergence of the MCMC procedure was assessed from the effective sample size scores (all > 100) using MrBayes. The first 25% of the trees were discarded as burn-ins. The remainder was used to calculate the posterior probabilities (PPs) for individual branches. The phylogenetic tree was visualized in FIGTREE v.1.4.3 (Rambaut 2012). All analyses were run on the CIPRES Science Gateway v 3.3 web portal (Miller et al. 2010).

**Table 1.** Taxa used in the phylogenetic analyses and their corresponding GenBank accession numbers.

Taxa	Strain number	GenBank Accession number		Reference
		ITS	β-tubulin	
*Allocryptovalsacastaneae*	CFCC52428	MW632945	MW656393	Zhu et al. (2021)
*Allocryptovalsacastaneicola*	CFCC52432	MW632947	MW656395	Zhu et al. (2021)
*Allocryptovalsacryptovalsoidea*	HVFIG02^T^	HQ692573	NA	Trouillas et al. (2011)
*Allocryptovalsaelaeidis*	MFLUCC150707	MN308410	MN340296	Konta et al. (2020)
*Allocryptovalsapolyspora*	MFLU 17-1218	NR153588	MG334556	Senwanna et al. (2017)
*Allocryptovalsarabenhorstii*	WA08CB	HQ692619	HQ692523	Trouillas et al. (2011)
*Allocryptovalsarabenhorstii*	GMB0416	OP935171	OP938733	Li et al. (2023)
*Allocryptovalsasichuanensis*	HKAS107017	MW240633	MW775592	Samarakoon et al. (2022)
*Allocryptovalsaxishuangbanica*	KUMCC21-0830	ON041128	ON081498	Maharachchikumbura et al. (2022)
*Allodiatrypealbelloscutata*	IFRD9100	OK257020	NA	Li et al. (2022)
*Allodiatrypearengae*	MFLUCC 15-0713	MN308411	MN340297	Konta et al. (2020)
*Allodiatrypeelaeidicola*	MFLUCC15-0737a	MN308415	MN340299	Konta et al. (2020)
*Allodiatrypeelaeidis*	MFLUCC150708a	MN308412	MN340298	Konta et al. (2020)
*Allodiatrypeeleiodoxae*	MFLU23-0357	OR571761	OR591484	Unpublished
*Allodiatrypedalbergiae*	MFLU23-0349	OR571759	OR771026	Unpublished
*Allodiatrypedalbergiae*	MFLU23-0350	OR571760	OR591487	Unpublished
*Allodiatrypetaiyangheensis*	IFRDCC2800	OK257021	OK345036	Li et al. (2022)
*Allodiatrypethailandica*	MFLUCC153662	KU315392	NA	Li et al. (2016)
*Allodiatrypetrigemina*	FCATAS842	MW031919	MW371289	Peng et al. (2021)
*Alloeutypa ﬂavovirens*	E48C	AJ302457	DQ006959	Rolshausen et al. (2006)
*Alloeutypamilinensis*	FCATAS4309	OP538689	OP557595	Ma et al. (2023)
*Anthostomadecipiens*	JL567	JN975370	JN975407	Luque et al. (2012)
*Cryptosphaerialigniota*	CBS273.87	KT425233	KT425168	Acero et al. (2004)
*Cryptosphaeriamulticontinentalis*	HBPF8	KT425178	NA	Trouillas et al. (2015)
*Cryptosphaeriapullmanensis*	ATCC52655	KT425235	KT425170	Trouillas et al. (2015)
*Cryptosphaeriapullmanensis*	HBPF24	KT425202	KT425137	Trouillas et al. (2015)
*Cryptosphaeriasubcutanea*	CBS240.87	KT425232	KT425167	Trouillas et al. (2015)
*Cryptovalsaampelina*	A001	GQ293901	GQ293972	Trouillas and Gubler (2010)
*Cryptovalsaampelina*	DRO101	GQ293902	GQ293982	Trouillas and Gubler (2010)
*Diatrypasimilisaustraliensis*	ATCC MYA-3540	NR111369	NA	Schoch et al. (2014)
*Diatrypebullata*	UCDDCh 400	DQ006946	DQ007002	Rolshausen et al. (2006)
*Diatrypecamelliae-japonicae*	GMB0427	OP935172	OP938734	Li et al. (2023)
*Diatrypedisciformis*	GNA14	KR605644	KY352434	Senanayake et al. (2015)
*Diatrypelancangensis*	GMB0045	MW797113	MW814885	Long et al. (2021)
*Diatrypemacowaniana*	Isolate D15C	AJ302431	NA	Acero et al. (2004)
*Diatrypequercicola*	CFCC-52418	MW632938	MW656386	Zhu et al. (2021)
*Diatryperubi*	GMB0429	OP935182	OP938740	Li et al. (2023)
*Diatrypellaatlantica*	HUEFS 194228	KM396615	KR363998	de Almeida et al. (2016)
*Diatrypellabanksiae*	CPC29118	KY173402	NA	Crous et al. (2013)
*Diatrypellabetulicola*	CFCC52411	MW632935	MW656383	Zhu et al. (2021)
*Diatrypelladelonicis*	MFLUCC15-1014	MH812994	MH812994	Hyde et al. (2019)
*Diatrypellaelaeidis*	MFLUCC15-0279	MN308417	MN340300	Konta et al. (2020)
*Diatrypellafatsiae-japonica*	GMB0422	OP935184	OP938744	Li et al. (2023)
*Diatrypellafrostii*	UFMGCB 1917	HQ377280	NA	Vieira et al. (2012)
*Diatrypellaheveae*	MFLUCC17-0368	MF959501	MG334557	Senwanna et al. (2017)
*Diatrypellahubeiensis*	CFCC52413	MW632937	NA	Zhu et al. (2021)
*Diatrypellairanensis T*	KDQ18	KM245033	KY352429	Mehrabi et al. (2015)
*Diatrypellalongiasca T*	KUMCC 20-0021	MW039349	MW239658	Dissanayake et al. (2021)
*Diatrypellamacrospora T*	KDQ15	KR605648	KY352430	Mehrabi et al. (2016)
*Diatrypellamajor*	ANM1947	KU320613	NA	de Almeida et al. (2016)
*Diatrypellapulvinata*	H048	FR715523	FR715495	de Almeida et al. (2016)
*Diatrypellatectonae*	MFLUCC120172b	KY283085	KY421043	Shang et al. (2017)
*Diatrypellavulgaris*	HVFRA02	HQ692591	HQ692503	Trouillas et al. (2011)
*Diatrypellayunnanensis*	VT01	MN653008	MN887112	Zhu et al. (2021)
*Eutypaarmeniacae*	ATCC28120	DQ006948	DQ006975	Rolshausen et al. (2006)
*Eutypaastroidea*	CBS292.87	DQ006966	DQ006966	Rolshausen et al. (2006)
*Eutypacamelliae*	HKAS107022	MW240634	MW775593	Samarakoon et al. (2022)
*Eutypacamelliae*	GZAAS24-0013	PP528182	PQ301430	This study
*Eutypacamelliae*	HKAS-107022	NR175674	MW775593	This study
*Eutypacerasi*	GMB0048	MW797104	MW814893	Long et al. (2021)
*Eutypaconsobrina*	F091	AJ302447	KY111596	Acero et al. (2004)
*Eutypacrustata*	CBS210.87	AJ302448	DQ006968	Rolshausen et al. (2006)
*Eutypalaevata*	CBS291.87	AJ302449	NA	Acero et al. (2004)
*Eutypalata*	EP18	HQ692611	HQ692611	Trouillas et al. (2011)
*Eutypalejoplaca*	CBS248.87	DQ006922	DQ006974	Rolshausen et al. (2006)
*Eutypamaura*	CBS219.87	DQ006926	DQ006967	Rolshausen et al. (2006)
*Eutypamicroasca*	BAFC51550	KF964566	KF964572	Grassi et al. (2014)
*Eutypapetrakii var. hedarae*	BENT014	OP038000	OP079836	Unpublished
*Eutypasparsa*	38023b	AY684220	AY684201	Trouillas and Gubler (2004)
*Eutypatetragona*	CBS284.87	DQ006923	DQ006960	Rolshausen et al. (2006)
*Eutypellacerviculata*	EL59C	AJ302468	NA	Acero et al. (2004)
*Eutypellamotuoensis*	FCATAS4082	OP538693	OP557599	Ma et al. (2023)
*Eutypellapersica*	IRAN 2540C	KX828144	KY352451	Mehrabi et al. (2019)
*Eutypellaquercina*	IRAN2543C	KX828139	KY352449	Mehrabi et al. (2019)
*Eutypellasemicircularis*	MP4669	JQ517314	NA	Mehrabi et al. (2016)
*Eutypellavirescens*	CBS205.36	MH855778	MH867286	Vu et al. (2019)
*Halocryptovalsasalicorniae*	MFLUCC 15-0185	MH304410	MH370274	Dayarathne et al. (2020)
*Halodiatrypeavicenniae*	MFLUCC 150953	KX573916	KX573931	Dayarathne et al. (2016)
*Halodiatrypesalinicola*	MFLUCC 15-1277	KX573915	KX573932	Dayarathne et al. (2016)
*Kretzschmariadeusta*	CBS 826.72	KU683767	KU684190	U’Ren et al. (2016)
*Monosporascuscannonballus*	CMM3646	JX971617	NA	Unpublished
*Monosporascuscannonballus*	ATCC26931	FJ430598	NA	Unpublished
*Neoeutypellabaoshanensis*	HMAS255436	MH822887	MH822888	Phookamsak et al. (2019)
*Paraeutypellacitricolca*	MFLU23-0352	OR563996	NA	Unpublished
*Paraeutypellaguizhouensis*	KUMCC 20-0017	MW036142	MW239661	Dissanayake et al. (2021)
*Paraeutypellapseudoguizhouensis*	GMB0420	OP935186	OP938748	Li et al. (2023)
*Pedumisporarhizophorae*	BCC44877	KJ888853	NA	Klaysuban et al. (2014)
*Peroneutypaaquilariae*	KUNCC-2210817	NR185767	OP572195	(Du et al. 2022).
*Peroneutypaanomianthe*	KUNCC-2315540	PP584741	PQ046048	Dissanayake et al. (2024)
*Peroneutypaanomianthe*	MFLU-210242	OK393705	NA	de Silva et al. (2022)
*Peroneutypaalsophila*	CBS250.87	AJ302467	NA	Acero et al. (2004)
*Peroneutypacomosa*	BAFC393	KF964568	NA	Grassi et al. (2014)
*Peroneutypacurvispora*	HUEFS136877	KM396641	KM396641	de Almeida et al. (2016)
*Peroneutypadiminutiasca*	MFLUCC17-2144	MG873479	NA	Shang et al. (2018)
*Peroneutypadiminutispora*	HUEFS192196	KM396647	NA	de Almeida et al. (2016)
***Peroneutypaguizhouensis***	**GZAAS24-0087**	**PQ878089**	**PQ876910**	**This study**
***Peroneutypaguizhouensis***	**GZAAS24-0088**	**PQ878090**	**PQ876911**	**This study**
*Peroneutypahainanensis*	GMB0424	OP935179	OP938746	Li et al. (2023)
*Peroneutypahainanensis*	GMB0425	OP935180	OP938747	Li et al. (2023)
*Peroneutypahongheensis*	KUNCC-23-16753	PP584742	PP951427	Dissanayake et al. (2024)
*Peroneutypaindica*	NFCCI 4393	MN061368	MN431498	Dayarathne et al. (2020)
*Peroneutypakochiana*	F092	AJ302462	NA	Carmarán et al. 2006
*Peroneutypakunmingensis*	HKAS 113189	MZ475070	MZ475070	Phukhamsakda et al. (2022)
*Peroneutypaleucaenae T*	MFLU 18-0816	MW240631	MW775591	Samarakoon et al. (2022)
*Peroneutypalongiasca*	MFLUCC170371	MF959502	MG334558	Senwanna et al. (2017)
*Peroneutypamackenziei*	MFLUCC16-0072	KY283083	KY706363	Shang et al. (2017)
*Peroneutypamangrovei*	PUFD526	MG844286	MH094409	Phookamsak et al. (2019)
*Peroneutypanayariophyti*	MFLU 23-0077	OQ981955	OR019690	Unpublished
*Peroneutypapolysporae*	NFCCI4392	MN061367	MN431497	Dayarathne et al. (2020)
*Peroneutypaqianensis*	GMB0431	OP935177	NA	Li et al. (2023)
*Peroneutypaqianensis*	GMB0432	OP935178	NA	Li et al. (2023)
*Peroneutyparubiformis*	MFLU 17-1185	MG873477	MH316763	Shang et al. (2018)
*Peroneutypascoparia*	MFLUCC 17-2143	MG873477	NA	Shang et al. (2018)
***Peroneutypawanfenglinensis***	**GZAAS24-0021**	**PP852356**	**PQ301419**	**This Study**
***Peroneutypawanfenglinensis***	**GZAAS24-0022**	**PP852353**	**PQ301420**	**This Study**
***Peroneutypazhujiashanesis***	**GZAAS24-0023**	**PP852363**	**PQ301421**	**This Study**
***Peroneutypazhujiashanesis***	**GZAAS24-0024**	**PP852362**	**PQ301422**	**This Study**
*Pseudodiatrypehainanensis*	GMB0054	MW797111	MW814883	Long et al. (2021)
*Quaternariaquaternata*	CBS278.87	AJ302469	NA	Acero et al. (2004)
*Quaternariaquaternata*	GNF13	KR605645	NA	Mehrabi et al. (2016)
*Stromatolineagrisea*	GMB4508	PQ113921	PQ115209	Habib et al. (2024)
*Stromatolineaguizhouensis*	GMB4523	PQ113922	PQ115210	Habib et al. (2024)
*Stromatolineaguizhouensis*	GMB4515	PQ113923	PQ115211	Habib et al. (2024)
*Stromatolineahydei*	GMB4509	PQ113924	PQ115212	Habib et al. (2024)
*Stromatolinealinearis*	MFLUCC 15-0198	KU940149	MW775587	Habib et al. (2024)
*Stromatolineaxishuiensis*	GMB4522	PQ113928	PQ115216	Habib et al. (2024)
*Vasilyevacinnamomic*	GMB0418	OP935174	OP938737	Li et al. (2023)
*Xylariahypoxylon*	CBS 122620	AM993141	KX271279	Peršoh et al. (2009)

## ﻿Results

### ﻿Phylogenetic analyses

**Figure 1.**
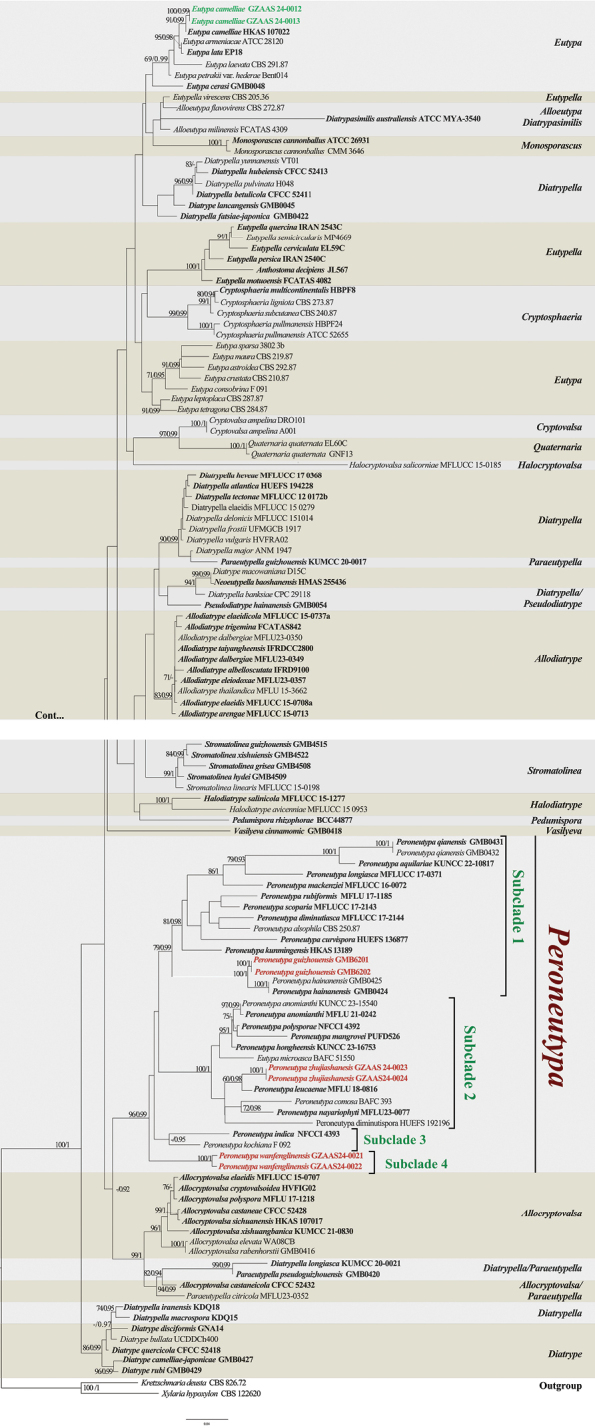
Phylogram generated from maximum likelihood analysis (RAxML) based on combined ITS and *tub2* sequences data. Bootstrap support values for maximum likelihood (ML) greater than 70% and Bayesian posterior probabilities (BPP) greater than 0.90 are displayed at the respective branches (ML/BPP). The newly described species are marked bold in red, and the new record is marked bold in green. Ex-type/type strains are indicated in black bold.

The combined ITS and *tub2* dataset consisted of 125 ingroup strains and two outgroups: *Kretzschmariadeusta* (CBS 826.72) and *Xylariahypoxylon* (CBS 122620). After the exclusion of ambiguously aligned regions and long gaps, the final combined data matrix contained 1,350 characters. The final ML optimization likelihood value of the best RAxML tree was −34243.792546. The tree topology derived from Maximum Likelihood (ML) analysis closely resembled that of Bayesian Inference (BI) analysis. The best-scoring RAxML tree is shown in Fig. 1.

The phylogenetic tree based on BI and ML approaches confirmed the position of our new species nested within the phylogenetic branch of the genus *Peroneutypa* (Fig. 1). According to the phylogenetic structure of the tree, *Peroneutypa* formed a large clade. However, the presence of *Eutypamicroasca* (BAFC 51550) in *Peroneutypa* clade renders its status polyphyletic. This suggests that the taxonomic status of *Eutypamicroasca* should be revisited for clarification. In the phylogram, the *Peroneutypa* clade is represented with 4 subclades. Subclade 1 includes 12 species, with the new species *P.guizhouensis* forming a sister relationship with *P.hainanensis*, supported strongly (ML/BI = 100/1). Subclade 2 contains 10 species, including the new species *P.zhujiashanesis*, which appears as a sister to *P.leucaenae* with moderate support (ML/BI = 60/0.99). Subclade 3 consists of two species, *P.indica* (NFCCI 4393) and *Peroneutypakochiana* (F092). Subclade 4 represents solely the new species *P.wanfenglinensis*, forming a distinct, well-supported clade (ML/BI = 96/0.99) at the basal position of the *Peroneutypa* clade. The newly generated sequences of *Eutypacamelliae* clustered with the type strain *E.camelliae* (HKAS107022). The sister branch to this clade includes *E.lata* (EP18) and *E.americana* (ATCC28120).

### ﻿Taxonomy

#### ﻿ Eutypa camelliae

Taxon classificationFungiXylarialesDiatrypaceae

Samarakoon, M.C & Hyde, K.D, Fungal Diversity 112 (1), 1–88 (2022)

1A39903F-4132-5229-BED1-642457447116

Index Fungorum: IF558718

Fig. 2

##### Description.

Saprobic on a dead branch of an unknown tree. Sexual morph: Stromata 3.1–8.3 mm diam., immersed in the bark, carbonaceous, effuse, confluent into irregularly elongated shape with diffuse margins, dark grayish to dull black surface, rarely with white dots on the surface, 20–50 loculate. Ascomata 390–620 µm in height, 190–340 µm in diameter (x̄ = 530 × 260 µm, n = 10), perithecia, coated with a white powdery substance in between, vary from globose to akin to an inverted flask, Ostiole slightly raised, conspicuous, 114–128 μm wide. Peridium 13.5–25 μm thick, two-layered, outer layer dark brown of textural angular cell, inner layer hyaline of elongated cell. Paraphyses septate, 3.5–7.2 μm (x̄ = 5.6 μm, n = 20) wide, constricted at the septa, longer than asci. Asci 50–120 × 4–6.6 μm (x̄ = 77.5 × 5.4 μm, n = 30), 8–spored clavate, with a rounded to truncate apex, J- apical rings. Ascospore 4–5.8 × 1.1–1.8 μm (x̄ = 4.93 × 1.35 μm, n = 30), overlap, allantoid, slightly curved, subhyaline, smooth, aseptate, often with a guttulae at both ends. Asexual morph: undetermined.

**Figure 2.**
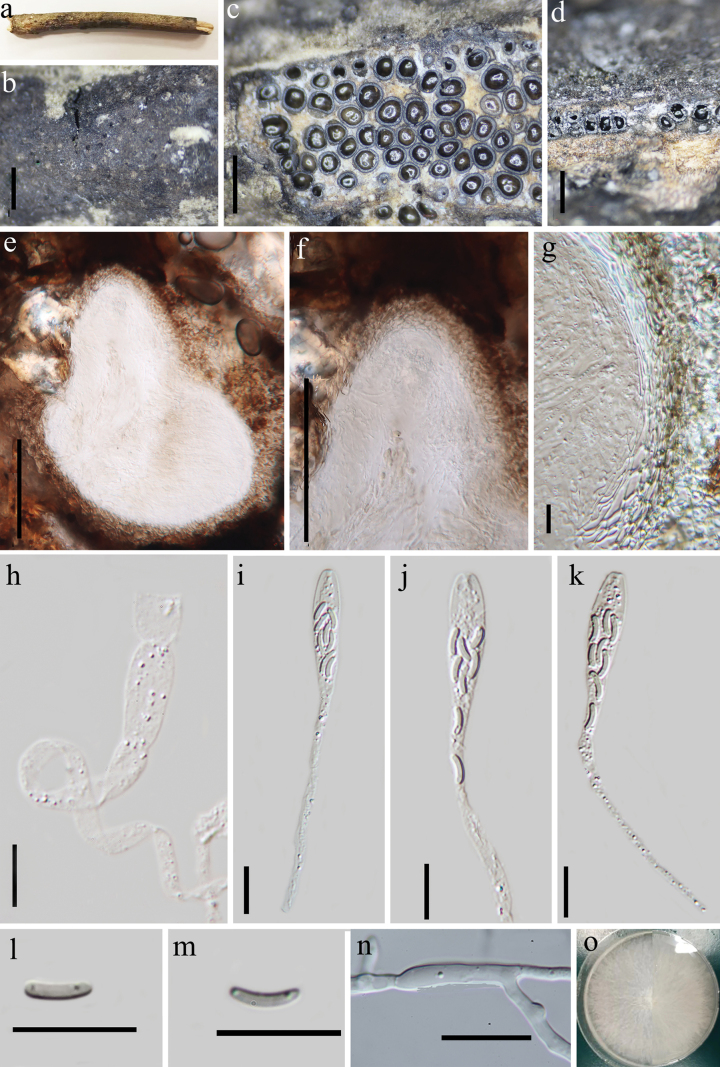
*Eutypacamelliae***a, b** stromata on dead branch **c** cross section of a stroma showing perithecia **d, e** vertical sections of ascomata **f** ostiole **g** peridium **h** paraphyses **i–k** asci **l, m** ascospores **n** germinating ascospore **p** culture on PDA. Scale bars: 1 mm (**a–d**); 100 μm (**e, f**); 10 μm (**i–o**).

##### Culture characteristics.

Colonies on PDA reach 60 mm in diameter after seven days at 28 °C. They are cottony, moderately dense, fluffy aerial mycelium, white from above and pale yellowish from below. Mycelium is composed of branched, septate, smooth-walled, hyaline hyphae.

##### Specimens examined.

China • Guizhou Province, Kaili City, Leigongshan State Reserve (108°11'47"E, 26°22'43"N), altitude 1664 m, on a dead branch of an unknown tree, 23 August 2023, Xin Y Mao & Y.Q. Kang, LGS19 (GZAAS24-0012, KUN-HKAS133146, strain number GZCC 24-0187). GenBank accession numbers (ITS: PP528179; *tub2*: PQ301429). Guizhou Province, Libo County, MaoLan National Nature Reserve (108°4'9"E, 25°17'8"N), altitude 694 m, on dead branches of an unknown tree, 22 March 2022, Xin Y Mao & Y.Q. Kang, LBML10 (GZAAS24-0013, KUN-HKAS133147; strain number GZCC 24-0188). GenBank accession numbers (ITS: PP528182; *tub2*: PQ301430).

##### Notes.

The sequence of our collection GZAAS24-0012 clustered with *Eutypacamelliae* in the phylogenetic tree, and ITS sequence BLAST searches also confirmed a 100% match with *E.camelliae* (HKAS 107022). The holotype description of this species was based on immature stromata, no asci or ascospores were observed in the material and the isolates were obtained from internal tissue of the stromata (Samarakoon et al. 2022). Our study represents the first report of *Eutypacamelliae* from China and provides the complete anatomical details, including mature stromata with asci and ascospores.

#### ﻿ Peroneutypa guizhouensis

Taxon classificationFungiXylarialesDiatrypaceae

X.Y. Mao, K. Habib & Y.Q. Kang sp. nov.

9FB5BF33-18F2-5610-AE41-22DCF6804E1C

Index Fungorum: IF903236

Fig. 3

##### Etymology.

The epithet refers to the name of the province from where the samples were collected.

##### Type.

China • Guizhou Province, Guiyang City, Panlongshan Forest Park. (106°49'18"E, 26°44'58"N), altitude 1242.1 m, on branch of an unidentified plant, 8 June 2024. Xin Y Mao & Y.Q. Kang, PLS29 (Holotype GZAAS24-0087; ex-type cultures GZCC 24-0296; Isotype KUN-HKAS 145344). GenBank accession numbers (ITS: PQ878089; *tub2*: PQ876910).

##### Description.

Saprobic on dead branches of an unidentified plant. Sexual morph: Stromata 0.5–1.5 mm in diameter, immersed in the host surface, ostiolar canals protruding through the bark, poorly developed, solitary, rarely gregarious, 1–4 locules, usually two, arranged irregularly, dark brown to black, glabrous, circular to irregular in shape, Ascomata (excluding neck) perithecia 400–720 μm high, 400–600 µm diam. (x̄ = 650 × 400 μm, n = 20), immersed in a stroma, black, globose to sub-globose, each has an individual ostiole with a long neck. Ostiolar canals: erumpent, smooth, 300–570 (x̄ = 435 μm) in length, cylindrical, smooth, curved at the apex. Peridium 48–56 μm (x̄ = 52.4 μm) thick, composed of two layers, outer layer dark brown to black, cells thick-walled, texture angularis, inner layers hyaline, cells flattened. Paraphyses 3–5.8 μm (x̄ = 4.9 μm, n = 20) wide, wider at the base, long, septate, smooth-walled. Asci 16–33 × 3.6–6.8 μm (x̄ = 24.1 × 5.0 μm, n = 30), unitunicate, 8-spored, clavate, apically truncates, with a J- apical ring. Ascospore 2.2–4.7 × 1.1–1.8 μm (x̄ = 3.3 × 1.4 μm, n = 30), overlapping, allantoid, subhyaline, smooth, aseptate, strongly curved, with 1–2 small guttules. Asexual morph: undetermined.

**Figure 3.**
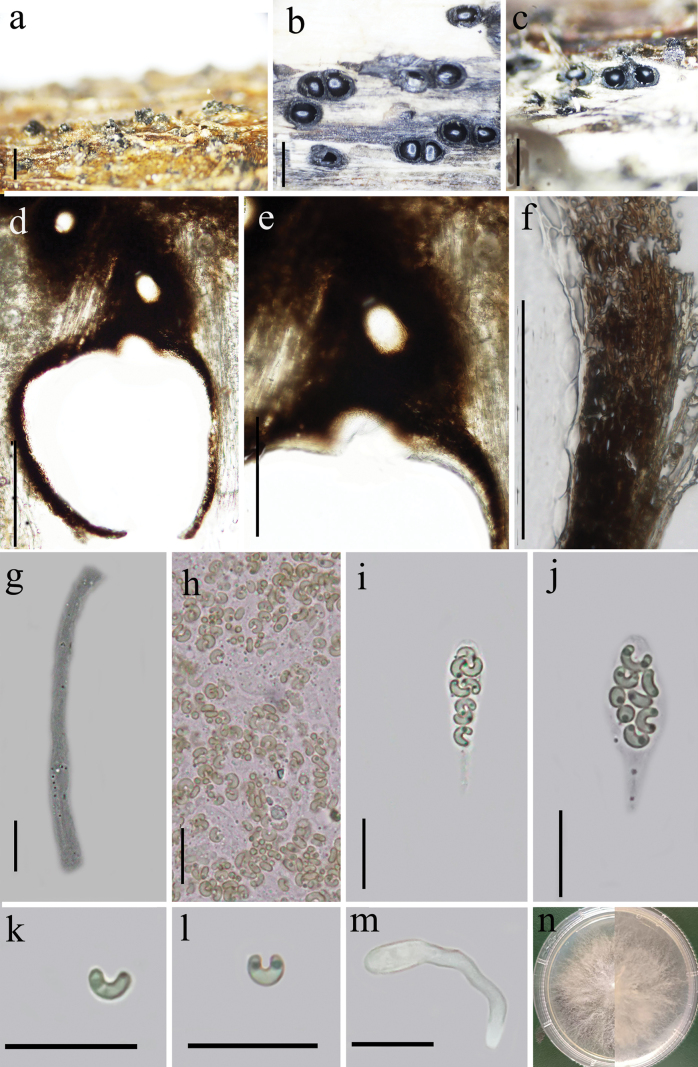
*Peroneutypaguizhouensis* (Holotype GZAAS24-0087) **a–c** stromata surface view **d** vertical section of ascomata **e** ostiole **f** peridium **g** paraphyses **h** numerous ascospores **i-j** asci **k, l** ascospores **m** germinating ascospore **n** culture on PDA. Scale bars: 1 mm (**a–d**); 100 μm (**e–g**); 10 μm (**h–o**).

##### Culture characteristics.

Colonies growing fast on PDA, reach 55 mm in 1 week at 28 °C, effuse, thin towards the edge, from above at first white, becoming dirty white at the edge after 2 weeks, from below brownish at the center, the rest white.

##### Additional specimens examined.

China • Guizhou Province, Zunyi county, Dashahe Natural Reserve (107°34'19"E, 29°7'32"N) altitude: 1900 m, on branches of an unidentified plant, 26 April 2024; Xin Y Mao & Y.Q. Kang, XHP01 (Paratype GZAAS24-0088, Isotype KUN-HKAS 145343, ex-paratype cultures GZCC 24-0297).). GenBank accession numbers (ITS: PQ878090; *tub2*: PQ876911).

##### Notes.

*Peroneutypaguizhouensis* is morphologically and phylogenetically like *P.hainanensis*, mainly due to its strongly curved ascospores. However, *P.guizhouensis* can be distinguished by its longer ostiolar necks (300–570 μm vs. 105–420 μm), smaller asci (16–33 μm in length, x̄ = 24.1 × 5.0 μm vs. 28.5–40 μm, x̄ = 33.5 × 5.5 μm), and significantly smaller ascospores (2.2–4.7 × 1.1–1.8 μm vs. 5.0–7.3 × 1–2 μm) (Li et al. 2023).

In addition to *Peroneutypahainanensis*, *P.guizhouensis* shares similarities with *P.diminutiasca*, *P.curvispora*, and *P.qianensis* due to its strongly curved ascospores.

Compared to *P.diminutiasca*, *P.guizhouensis* has significantly longer ostiolar necks (300–570 μm vs. 105–280 μm), a thicker peridium (48–56 μm vs. 15–32 μm), and smaller ascospores (2.2–4.7 × 1.1–1.8 μm vs. 3.1–5.9 × 1.3–2.2 μm) (Shang et al. 2018). *Peroneutypacurvispora* differs from *P.guizhouensis* in having much longer ostiolar necks (400–800 μm vs. 300–570 μm), smaller asci (9–16.5 × 4–6 μm vs. 16–33 μm), and the absence of paraphyses (vs. present) (de Almeida et al. 2016).

Compared to *P.qianensis*, *P.guizhouensis* differs in having longer ostiolar necks (300–570 μm vs. 105–420 μm), larger asci (16–33 × 3.6–6.8 vs. 16.5–20.5 × 4–6 μm), and smaller ascospores (2.2–4.7 × 1.1–1.8 μm vs. 4.5–6.3 × 1.5–0.3 μm) and presence of paraphyses (vs. lack) (Li et al. 2023).

These morphological differences (Table 2), combined with phylogenetic evidence, highlight the distinctiveness of *P.guizhouensis* and confirm its status as a new species.

**Table 2.** Comparison of new taxa with closely related species.

Species	Stromata (mm wide)	Ascomata (µm)	Ostiolar canal (µm)	Peridium (µm)	Paraphyses (μm)	Asci (µm)	Ascospores (µm)	Country	Host	References
***P.guizhouensis***	0.5–1.5	400–720 × 400–600	300–570 long	48–56	3–5.5	16–33 × 3.6–6.8, J– apical ring, short pedicellate	2.2–4.7 × 1.1–1.8, strongly curved	China	Unknown tree branch	This Paper
***P.wanfenglinensis***	1.9–2.5	330–620 × 280–520	120–140 long	20–45	3.5–6	20–30.5 × 3–5, J– apical ring, long pedicellate	3–4.2 × 1–2, slightly curved	China	*Betulaplatyphylla*	This Paper
***P.zhujiashanesis***	1–2.8	550–910 × 400–570 µm	50–145 long	24–42	3.5–6	23–31 × 3.5–7, J– apical ring, long pedicellate	3.5–5 × 1–1.5, slightly curved	China	Unknown tree branch	This Paper
***P.indica***	N/A	375 × 202	100–350 long,	15–35	1–2	42 × 3.5, short pedicellate, J– apical ring	5.5 × 1.3, slightly curved	India	*Suaedamonoica*	Dayarathne et al. 2020
***P.curvispora***	0.6–3	300–700	400–800 long	N/A	Absent	9–16.5 × 4–6, long pedicellate	3–5 × 1–2, strongly curved	Brazil	Unidentified plant	de Almeida et al. (2016)
***P.diminutiasca***	1.2–1.4	75–220 × 99–340	193 × 48	15–32	4–7	12–33 × 2.8–5, J− apical ring, long pedicellate,	4.2 × 1.7 µm, slight to moderately curved	China, Thailand	Unidentified wood	Shang et al. 2018; Du et al. 2022
***P.hainanensis***	0.4–0.7	350–600 × 130–300	105–420 × 80–120	45–65	N/A	28.5–40 × 3.5–6.5, J− apical ring	5.0–7.3 × 1–2, strongly curved	China	Unidentified plant	Li et al. 2023
***P.kochiana***	N/A	150	Neck not prominent	N/A	N/A	18–28 long, J+ apical ring	4.5–6 × 1.5–2 slightly curved	Russia, Spain	*Atriplexhalimus*	Acero et al. 2004; Carmarán et al. 2006
***P.leucaenae***	N/A	655 × 525	275–350 long	22–43	3.2–7 wide, septate	33 × 4.2, J+ apical ring, long pedicellate	2.9–3.7 × 0.9–1.3 slightly curved	Thailand	*Leucaenaleucocephala*	Samarakoon et al. 2022
***P.qianensis***	1.5–2	320–540 × 175–290	105–420 × 80–120	45–65	N/A	16.5–20.5 × 4–6, J− apical ring	4.5–6.3 × 1.5–0.3, slightly curved	China	Unidentified plant	Li et al. 2023

#### ﻿ Peroneutypa wanfenglinensis

Taxon classificationFungiXylarialesDiatrypaceae

X.Y. Mao, K. Habib & Y.Q. Kang sp. nov.

D0C38D89-232E-59CC-83D1-F5C46A1EFB5D

Index Fungorum: IF902676

Fig. 4

##### Etymology.

The epithet refers to the name of the location (Wan Feng Lin State Reserve), where the type specimen was collected.

##### Type.

China • Guizhou Province, Xingyi City, Wan Feng Lin State Reserve (104°55'28"E, 24°59'26"N), altitude 896.8 m, on dead branches of *Betulaplatyphylla*, 28 May 2022, Xin Y Mao & Y.Q. Kang, WFL02 (Holotype GZAAS24-0021; Isotype KUN-HKAS133157, ex-type cultures GZCC 24-0196). GenBank accession numbers (ITS: PP852356; *tub2*: PQ301419).

##### Description.

Saprobic on decaying branches of *Betulaplatyphylla*. Sexual morph: Stromata 1.90–2.5 mm in diameter, interior, solitary to gregarious, with 1–4 perithecia, immersed, erumpent by a long ostiolar canal, dark brown to black, surface glabrous, shape circular to irregular, arranged irregularly. Ascomata (excluding necks) 330–620 μm high, 280–520 µm diam. (x̄ = 540 × 320 μm, n = 10), immersed in a stroma, black, monostichous to distichous, circular to oval, each has an individual ostiole with a short neck. Ostiolar canals erumpent, smooth, 120–140 μm (x̄ = 135 μm, n = 10) long, arch-shaped, sulcate, and curved at the apex. Peridium 20–45 μm (x̄ = 31.85 μm) thick, composed of two layers, outer layer brown to dark, cells thick-walled, texture angularis, inner layers hyaline, cells flattened, texture angularis. Paraphyses septate, slightly swollen at the septa, 3.5–6 μm (x̄ = 5.4 μm, n = 20) wide. Asci 20–30.5 × 3–5 μm (x̄ = 25 × 4 μm, n = 30), unitunicate, 8-spored, clavate, with apically rounded to truncate ends, with a J- apical ring. Ascospore 3–4.2 × 1–2 μm (x̄ = 3.6 × 1.4 μm, n = 30), overlapping, allantoid, subhyaline, smooth, aseptate, with 1–2 oil droplets. Asexual morph: undetermined.

**Figure 4.**
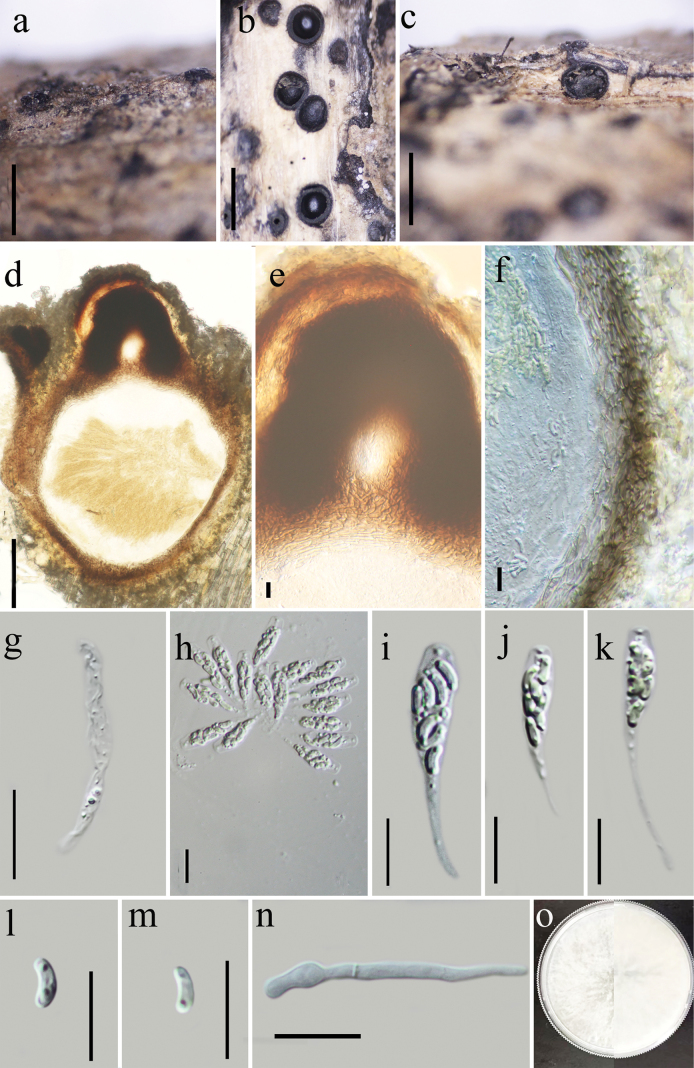
*Peroneutypawanfenglinensis* (Holotype GZAAS24-0021) **a** surface view of stromata **b** cross section of a stroma showing perithecia **c, d** vertical sections of ascomata **e** ostioles **f** peridium **g** paraphyses **h–k** asci **l, m** ascospores **n** germinating ascospore **o** culture on PDA. Scale bars: 1 mm (**a–c**); 10 μm (**d, e**); 100 μm (**f–o**).

##### Culture characteristics.

Colonies growing fast on PDA, reach 60 mm in 1 week at 28 °C, effuse, velvety to hairy, nearly circular, dense towards the edge, fluffy aerial mycelium, appear white from above and pale from below. Mycelium is composed of branched, septate, smooth-walled, hyaline hyphae.

##### Additional specimens examined.

China • Guizhou Province, Zunyi City, Chishui Zhuhai National Forest Park (105°99'14"E, 28°47'19"N), altitude 838 m, on branches of an unidentified plant, 21 July 2023. Xin Y Mao, CSZH01 (Paratype GZAAS24-0022; KUN-HKAS133156; ex-paratype cultures, GZCC 24-0197). GenBank accession numbers (ITS: PP852353; *tub2*: PQ301420).

##### Notes.

BLAST results reveal that *Peroneutypawanfenglinensis* is closely related to *P.kochiana*. However, *P.wanfenglinensis* differs morphologically from *P.kochiana* in having smaller ascospores (3–4.2 × 1.0–1.9 μm vs. 4.5–6 × 1.5–2 μm), larger ascomata (330–620 μm high, 280–520 μm diam. vs. 150 μm diam.), and asci with a J- (non-amyloid) apical ring, compared to the J+ (amyloid) apical ring in *P.kochiana* (Carmarán et al. 2006). Sequence analysis also indicates a notable difference between *P.wanfenglinensis* and *P.kochiana*, showing a relatively low ITS similarity of 91%.

In terms of ascomata size and apical ring, *Peroneutypawanfenglinensis* is more like *P.indica*. However, their ascus and ascospore dimensions can differentiate the two species. *Peroneutypaindica* has longer asci (35–47 μm vs. 20–30.5 μm) and ascospores (4–8 μm vs. 3–4.2 μm) compared to *P.wanfenglinensis* (Dayarathne et al. 2020).

In terms of ascomata and ascospore dimensions, *Peroneutypawanfenglinensis* is comparable to *P.leucaenae*. However, *P.leucaenae* can be distinguished by its significantly longer ostiolar neck (275–350 μm vs. 120–140 μm) and larger asci (average 33 × 4.2 μm vs. average 25 × 4 μm). Additionally, *P.leucaenae* is characterized by a J+ (amyloid) apical ring, contrasting with the J− (non-amyloid) apical ring observed in *P.wanfenglinensis* (Du et al. 2022).

These distinct morphological features (Table 2), together with their distinct phylogenetic position, support the recognition of *P.wanfenglinensis* as a new species.

#### ﻿ Peroneutypa zhujiashanesis

Taxon classificationFungiXylarialesDiatrypaceae

X.Y. Mao & Y.Q. Kang sp. nov.

1DDA7BA4-3998-5723-914F-4CAD015F4B86

Index Fungorum: IF902672

Fig. 5

##### Etymology.

The epithet refers to the name of the location where the type specimen was collected, Zhujiashan National Forest Park.

##### Type.

China • Guizhou Province, Douyun City, Weng‘an County, Zhujiashan National Forest Park (107°38'35"E, 26°58'35"N), altitude 848 m, on branches of an unidentified plant, 14 February 2022. Xin Y Mao & Y.Q. Kang, ZJS14 (Holotype GZAAS24-0023; KUN-HKAS133155; ex-type GZCC 24-0198). GenBank accession numbers (ITS: PP852363; *tub2*: PQ301421).

##### Description.

Saprobic on decaying branches of an unknown tree. Sexual morph: Stromata 1–2.8 mm in diameter, immersed in the host surface, with necks conspicuously protruding through the bark, erumpent through an ostiolar canal, solitary to gregarious,1–3 locules, mostly solitary, arranged irregularly, dark brown to black, glabrous, circular to irregular in shape, arranged irregularly, delimited by a black zone in host tissues. Ascomata (excluding neck) immersed in a stroma, dark brown to black, perithecia 550–910 μm high, 400–570 µm diam. (x̄ = 790 × 520 μm, n = 10), black, single to aggregated, globose to sub-globose, each has an individual ostiole with a long neck. Ostiolar canals erumpent, smooth, 50–145 μm (x̄ = 134.69 μm) in length, cylindrical, and curved at the apex. Peridium 24–42 μm (x̄ = 32.73 μm) thick, composed of two layers, outer layer dark brown to black, cells thick-walled, texture angularis, inner layers hyaline, cells flattened, texture angularis. Paraphyses 3.5–6 μm (x̄ = 5.4 μm, n = 20) wide, wider at the base, long, septate, smooth-walled, constricted at septa. Asci 23–31 × 3.5–7 μm (x̄ = 27.5 × 5.5 μm, n = 30), unitunicate, 8-spored, clavate, apically truncates, with a J- apical ring. Ascospore 3.5–5 × 1–1.5 μm (x̄ = 4.2 × 1.3 μm, n = 30), overlapping, allantoid, subhyaline, smooth, aseptate, with 1–2 small guttules. Asexual morph: undetermined.

**Figure 5.**
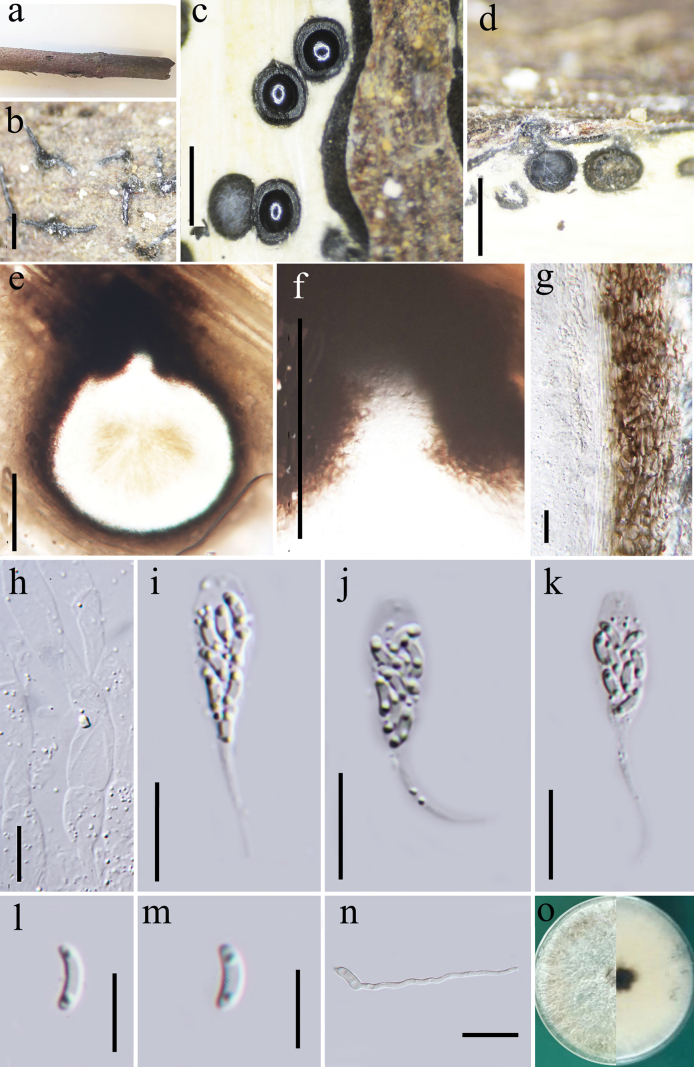
*Peroneutypazhujiashanesis* (Holotype GZAAS24-0023) **a, b** stromata on dead branch **c** transverse section of ascomata **d, e** vertical section of ascomata **f** ostioles **g** peridium **h** paraphyses **i–k** asci **l, m** ascospores **n** germinating ascospore **o** culture on PDA. Scale bars: 1 mm (**a–d**); 100 μm (**e–f**); 5 μm (**i–o**).

##### Culture characteristics.

Colonies grow fast on PDA, reach 60 mm in 1 week at 28 °C, effuse towards the edge, from above at first white, becoming dirty white at the edge after 2 weeks, from below black at center, the rest white.

##### Additional specimens examined.

China • Guizhou Province, Anlong county, Xianheping National Forest Park (105°36'26"E, 24°58'39"N) altitude: 1298 m, on branches of an unidentified plant, 30 May 2022; Xin Y Mao & Y.Q. Kang, XHP01 (Paratype GZAAS24-0024; KUN-HKAS-133158; ex-paratype GZCC 24-0199). GenBank accession numbers (ITS: PP852362; *tub2*: PQ301422).

##### Notes.

Phylogenetically, *Peroneutypazhujiashanesis* is closely related to *P.leucaenae*. Morphologically, it also shares similarities with *P.leucaenae* in terms of ascomata size and the shape and size of paraphyses. However, *P.zhujiashanesis* can be distinguished from *P.leucaenae* by its smaller asci (23–31 × 3.5–7 μm vs. 30–37 × 3.8–4.5 μm) and longer ascospores (3.5–5 μm vs. 2.9–3.7 μm) (Samarakoon et al. 2022). Additionally, *P.zhujiashanesis* has smaller ostiolar necks (50–145 μm) and a J– apical ring, whereas *P.leucaenae* has longer ostiolar necks (275–350 μm) and an amyloid apical ring.

Morphologically, *Peroneutypazhujiashanesis* is also like *P.diminutiasca* in ascospore size and presence of a J– subapical ring. However, *P.diminutiasca* differs by having smaller ascomata (147–218 μm in diameter), with longer ostiolar neck (average 193 μm vs 134.6 μm), and possessing 1–10 locules per ascomata (vs 1–3 loculate, mostly single) (Du et al. 2022).

Based on these morphological differences (Table 2) and phylogenetic evidence, we introduce our collection as a new species, *Peroneutypazhujiashanesis*.

## ﻿Discussion

The taxonomy of the Diatrypaceae has long been challenging, with unstable generic boundaries that lack strong morphological or phylogenetic support. Current classifications often fail to reflect the evolutionary relationships among these fungi accurately. Our phylogenetic analyses, based on ITS and β-tubulin sequences, corroborate previous findings (Shang et al. 2017; Dissanayake et al. 2021; Zhu et al. 2021; Long et al. 2021; Li et al. 2023) and reveal that several genera, such as *Cryptosphaeria*, *Diatrype*, *Diatrypella*, and *Eutypa*, are not monophyletic and contain multiple problematic clades.

The phylogeny of the family reveals many clades that may represent distinct genera, suggesting that the current classification is too simplistic. A comprehensive revision involving extensive sampling and a combination of taxonomic methods (integrating morphology, molecular data, chemical profiles and genomic information) is essential to resolve these complex relationships and achieve a more natural classification for the Diatrypaceae. *Peroneutypa* exemplifies the unresolved taxonomic and phylogenetic issues that are common within Diatrypaceae.

Phylogenetically, *Peroneutypa* species form a well-supported clade (Fig. 1). However, the inclusion of *Eutypellamicroasca* within this clade suggests its polyphyletic nature. In our investigation, we found that molecular data did not correlate well with morphological characteristics. Species that are phylogenetically close differed significantly in morphology. For instance, *P.polysporae*, characterized by multispored asci, clustered with *P.mangrovei*, which possesses eight-spored asci. Similarly, the newly described species *Peroneutypaguizhouensis* clustered with *P.hainanensis*, both of which possess strongly curved ascospores. However, other strongly curved ascospore-bearing species, such as *P.curvispora*, *P.diminutiasca*, *P.obesa*, and *P.qianensis*, are phylogenetically distant. Another example is *Peroneutypazhujiashanesis*, which has a J- apical ring, clustering with *P.leucaenae*, a species that possesses a J+ apical ring. Only six species in the genus are known to have a J+ apical ring (*P.alsophila*, *P.comosa*, *P.exigua*, *P.iranica*, *P.kochiana*, and *P.leucaenae*), and they are not closely clustered phylogenetically.

These analyses suggest that the genus *Peroneutypa* exhibits a complex evolutionary history, where phylogenetic relationships are not always reflected in morphological traits. The discordance between molecular and morphological data underscores the need for a comprehensive integrative approach. The use of additional genetic regions, such as LSU, SSU, *rpb2*, and *tef-1α*, should be explored to achieve more accurate phylogenetic analyses. Coupled with detailed morphological studies, these efforts will enable the precise delineation of species boundaries and provide deeper insights into the evolutionary relationships within this genus. The inclusion of newly described species in future studies, such as those introduced in this research, will continue to refine the phylogeny and enhance understanding of the genus’s diversity and evolutionary patterns, and contribute to a more robust and accurate classification system.

*Peroneutypa* species are mostly reported from plant species belonging to the families Thymelaeaceae, Rubiaceae, Moraceae, Fabaceae, and Euphorbiaceae, among others (Du et al. 2022) and primarily associated with woody angiosperms, particularly trees and shrubs, and are rarely found on herbaceous plants. To date, there are no reports of *Peroneutypa* species occurring on gymnosperms. Among angiosperms, *Peroneutypa* species are mainly recorded on dicots, with the exception of *P.scoparia* and *P.bellula*, which have been documented on monocots.

Within this genus, species can be distinguished based on several morphological traits, including the size of the ascomata, ostiolar canal length, asci, and ascospores. Additional characteristics, such as the shape of the asci and ascospores, the reaction of the ascus apex in Melzer’s reagent, and the presence or absence of paraphyses, are also used in differentiating species (Shang et al. 2017; Du et al. 2022). *Peroneutypapolymorpha* and *P.rubiformis* are unique within the genus for having larger asci, measuring more than 40 µm in length. Most other species in the genus have smaller asci. Similarly, ascospore size is generally consistent across the genus, ranging from 3–6 × 1–2 µm, except *P.polysporae*, which has notably larger ascospores measuring up to 9 × 1.8 µm.

Ostiolar canal length is another key characteristic for species delimitation. Species such as *P.coffea*, *P.comosa*, *P.curvispora*, *P.cylindrica*, *P.cyphelioides*, *P.exigua*, *P.komonoensis*, *P.macroceras*, *P.philippinarum*, and *P.variabilis* all have longer ostiolar canals (> 500 µm), distinguishing them from other species in the genus that have shorter canals (< 400 µm). Curved ascospores have been observed in *P.curvispora*, *P.diminutiasca*, *P.hainanensis*, *P.obesa* and *P.qianensis*, another prominent differentiating feature within the species.

Overall, the morphological variability in *Peroneutypa* requires using a combination of these traits for accurate species identification.

## Supplementary Material

XML Treatment for Eutypa camelliae

XML Treatment for Peroneutypa guizhouensis

XML Treatment for Peroneutypa wanfenglinensis

XML Treatment for Peroneutypa zhujiashanesis

## ﻿Additional information

### Author ORCIDs

Xinying Mao https://orcid.org/0000-0003-0684-3124

Kamran Habib https://orcid.org/0000-0003-2572-0306

### Data availability

The datasets generated during and/or analyzed during the current study are available in the MycoBank repository (included in the manuscript) and GenBank (included in Table 1). Also, the datasets generated during and/or analyzed during the current study are available from the corresponding author upon reasonable request.
